# Characterising global risk profiles of Mpox clade Ib importation

**DOI:** 10.1093/jtm/taae136

**Published:** 2024-10-16

**Authors:** Toshiaki R Asakura, Sung-mok Jung, Shihui Jin, Gang Hu, Akira Endo, Borame Lee Dickens

**Affiliations:** Department of Infectious Disease Epidemiology and Dynamics, London School of Hygiene & Tropical Medicine, London WC1E 7HT, UK; Centre for Mathematical Modelling of Infectious Diseases, London School of Hygiene & Tropical Medicine, London WC1E 7HT, UK; School of Tropical Medicine and Global Health, Nagasaki University, Nagasaki 852-8523, Japan; Carolina Population Center, University of North Carolina at Chapel Hill, Chapel Hill, NC 27516, USA; Saw Swee Hock School of Public Health, National University of Singapore, 117549, Singapore; Saw Swee Hock School of Public Health, National University of Singapore, 117549, Singapore; Department of Infectious Disease Epidemiology and Dynamics, London School of Hygiene & Tropical Medicine, London WC1E 7HT, UK; Centre for Mathematical Modelling of Infectious Diseases, London School of Hygiene & Tropical Medicine, London WC1E 7HT, UK; School of Tropical Medicine and Global Health, Nagasaki University, Nagasaki 852-8523, Japan; Saw Swee Hock School of Public Health, National University of Singapore, 117549, Singapore; Saw Swee Hock School of Public Health, National University of Singapore, 117549, Singapore

**Keywords:** Mpox, clade Ib, importation risk, travel volume

## Abstract

The novel mpox clade Ib initially identified in the Domestic Republic of Congo has spread to its multiple neighbouring countries as well as countries beyond the African continent. We characterised the global risk of importation of mpox clade Ib, highlighting the need to ramp up surveillance capacity for early detection.

In September 2023, a cluster of mpox cases were reported in the Kamituga health zone of the South Kivu province, the Democratic Republic of Congo (DRC), from which a novel virus clade Ib was identified.^1^ Genetic analyses have shown evidence of sustained human-to-human transmission since its emergence,^1^ with rapid spread to other health zones in South Kivu and neighbouring countries, including Burundi, Rwanda, Uganda and Kenya by August 2024. Unlike clade Ia, primarily driven by zoonotic exposure and limited human-to-human transmission typically within households,^2^ clade Ib has been reported to be additionally spread through sexual contact, highlighted by the increased risk among sex workers and their clients.^3^ Although substantial uncertainty exists in whether and how clade Ib may have altered its behaviour, the observed shift in transmission modes and rapid spread has prompted the (re-)declaration of the World Health Organisation’s Public Health Emergency of International Concern (PHEIC) on mpox. Shortly after, clade Ib reached beyond the African continent with two travel-associated cases reported in Sweden and Thailand on 15 and 22 August 2024, respectively.^4^

International flight travel volume data can help to infer and contextualize the country-specific risk of importing clade Ib mpox cases into countries currently without local transmission (Figure 1A). We used a simple statistical model assuming the risk of importation in a country is proportional to the OAG flight travel volume (monthly average from May 2023 to June 2024) from the source countries. Given the two importations observed outside Africa, we estimate that 23 183 (95% confidence interval: 3765-72 255) local clade Ib infections may have accumulated in the DRC, assuming the DRC is the primary source of exportation and that no additional undetected importations exist (see Supplementary Materials for methodological details and Table S1 for a sensitivity analysis assuming additional source countries). As of August 2024, the reported numbers of confirmed and suspected cases in the North and South Kivu provinces—where clade Ib is known to circulate in the DRC—are 1443 and 5077, respectively. This discrepancy may suggest moderate underreporting of clade Ib infections in the DRC.

**Figure 1:**
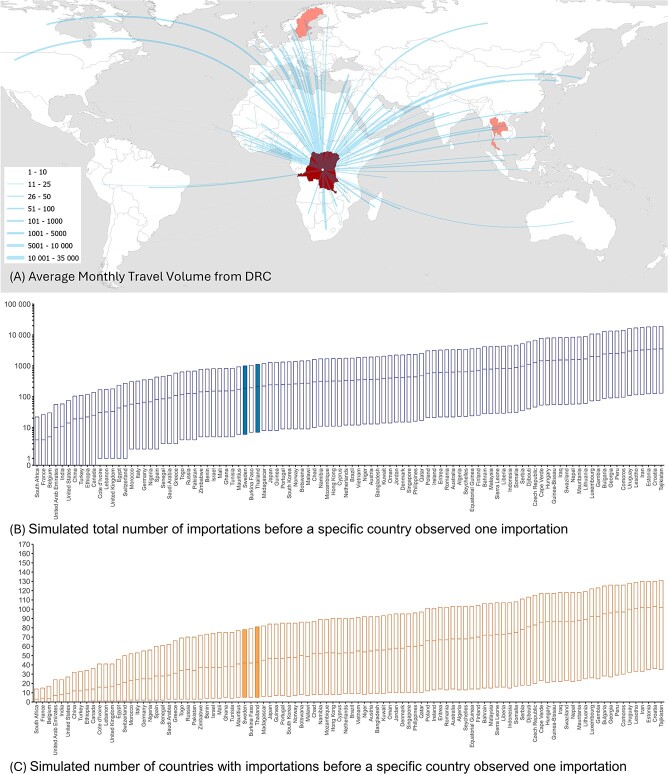
Travel volumes and simulated number of importations and countries before the first importation in a specific country. (A) Travel volume from the DRC to international countries. (B) Simulated total number of importations before a specific country observed one importation and (C) Simulated number of countries with importations before a specific country observed one importation. Only the top 100 countries with the highest travel volume are included in the figure. Sweden and Thailand reporting an imported clade Ib case are highlighted. This analysis assumes that the DRC is the only source of exportation while sensitivity analyses assuming the additional countries as sources (including Burundi, Uganda, Kenya and Rwanda) are available in the Supplementary Materials (Figures S1 and S2). Flight volumes for the above countries to Angola, Burundi, Cameroon, DRC, Central African Republic, Gabon, Kenya, Rwanda, Republic of the Congo, South Sudan, Tanzania, Uganda and Zambia were excluded as they have reported historic or current clade Ia or Ib mpox cases.

We simulated the likely phase at which a country may experience its first clade Ib importation (Figure 1B and C) by estimating how many imported cases and countries would be recorded before the first importation occurs. This provides an overview of the clade Ib importation landscape, showing a likelihood of immediate importation in a country based on importations reported elsewhere. We obtained similar results in our sensitivity analysis where we assumed countries with confirmed cases other than the DRC are also sources of exportation (Figures S1 and S2).

Our simulations suggest that neither Sweden (36th [95% range: 3rd–74th] country to import in our simulation) nor Thailand (47th [6th–88th]), reporting the first two importations, is among the countries to expect the earliest importation. While the full travel history of these two cases, including origin, destination and stopovers, may explain this discrepancy, it also suggests potential undetected importations in countries with high travel volume. Additionally, clade Ib’s deletion in the target domain could have affected PCR testing accuracy for the clade differentiation from globally circulating clade IIb,^5^ especially before the PHEIC declaration. Enhancing surveillance capacity worldwide to detect possible clade Ib arrivals is crucial to prevent further international spread, which would also allow the global health community to sustain support for containment in the most affected region.

## Supplementary Material

Importation_analysis_mpox1b_suppl_20240921_taae136

## Data Availability

The data analysis code in the present study is deposited on https://github.com/toshiakiasakura/mpox1b_importation_risk. The OAG flight volume data was not uploaded as per the terms of the contract.
