# Supplementary material for: Characterising global risk profiles of Mpox clade Ib importation
Source: J Travel Med. 2024 Oct 16;31(8):taae136. doi: 10.1093/jtm/taae136 (PMC11646081; doi:10.1093/jtm/taae136)
Supplement: Importation_analysis_mpox1b_suppl_20240921_taae136 [file importation_analysis_mpox1b_suppl_20240921_taae136.pdf]

Supplementary information for

Characterising global risk profiles of Mpox clade Ib importation

Toshiaki R. Asakura, MD, MSc, Sung-mok Jung, PhD, Shihui Jin, PhD, Gang Hu, PhD, Akira Endo, MD, PhD, Borame Lee Dickens, PhD

Contents

- Material and methods
    - Data source
    - Back-projecting the cumulative incidence in countries with ongoing spread
    - Model for the simulated total number of importations and simulated number of countries ever importing mpox clade Ib
- Supplementary results
    - Back-projected cumulative incidence in countries with ongoing outbreaks
    - Sensitivity analysis for the simulated number of importations and number of countries ever importing given one observation in a specific country
- Table S1
- Figure S1-S2
- References

Material and methods

Data source

We used the OAG flight volume dataset spanning from May 2023 to June 2024, which provides comprehensive information on flight volumes between individual airports worldwide.[1] In the present study, we aggregated airport data to obtain country-specific flight volumes and took the monthly average over the included months. The flight volume between any two countries includes direct flights and indirect flights that involve transit through third countries.

Population size data for the Democratic Republic of Congo (DRC), Burundi, Rwanda, Uganda, and Kenya were retrieved from the United Nations population data for 2024.[2]

Back-projecting the cumulative incidence in countries with ongoing spread

We estimated the cumulative incidence of mpox clade Ib in DRC at the time of importation events in Sweden and Thailand. We assumed that the probability of contracting an infection in DRC is equivalent between travellers and residents. Let C_t be the cumulative incidence of mpox clade Ib in DRC by day t and $O_{i,t}$ be the cumulative incidence in an importing country i by day t. We model $O_{i,t}$ to follow a binomial process:

$$O_{i,t} \sim \mathrm{Bin}(C_t, p_i), \qquad (S1)$$

where p_i is the proportion of travellers in DRC and subsequently moving to country i by plane among the population of DRC (N_{DRC}). This proportion (p_i) is described by

$$p_i = \frac{m_{DRC,i}}{30 N_{DRC}} T_d, \qquad (S2)$$

where $m_{DRC,i}$ is the average monthly flight volume from DRC to country i and T_d is the average length of stay in DRC (assumed to be 7 or 10 days).

Even though DRC experienced mpox outbreaks across multiple provinces, the majority of mpox clade Ib were reported specifically from South Kivu province. However, Equation S2 still applies even if the source of clade Ib importation was almost limited to South Kivu, as long as we can assume the prevalence of travellers is distributed to South Kivu and other provinces within DRC proportionally to their population sizes. That is, the assumed proportionality ensures $\frac{m_{DRC,i}}{N_{DRC}} = \frac{m_{SK,i}}{N_{SK}}$, which holds Equation S2 invariant.

The likelihood function for the cumulative incidence is then described by

$$L(C_t) = \prod_i \binom{C_t}{O_{i,t}} p_i^{O_{i,t}} (1 - p_i)^{C_t - O_{i,t}}, \qquad (S3)$$

We included countries with the top 100 flight volumes from DRC, while we excluded the following countries which have reported historic or current clade I mpox cases[3]: Angola, South Suda, Tanzania, Zambia, Republic of the Congo, Central African Republic, Burundi, Rwanda, Uganda, Kenya, Cameroon, and Gabon.

Since both of the two imported cases in Sweden and Thailand visited multiple African countries, we conducted two sensitivity analyses, assuming the primary source of exportation was i) DRC and Burundi, or ii) DRC, Burundi, Rwanda, Uganda and Kenya. The combined monthly average flight volume and combined population size for those countries were used in Equation S2. For example, p_i for DRC and Burundi was described by

$$p_i = \frac{m_{DRC,i} + m_{BI,i}}{30(N_{DRC} + N_{BI})} T_d, \tag{S4}$$

where $m_{BI,i}$ is the average monthly outgoing flight volume from Burundi to country i and N_{BI} is the population size in Burundi.

We estimated C_t using the maximum likelihood estimation with the differential evolution for the optimisation algorithm. The 95% confidence interval was obtained using the profile likelihood method.

Model for the simulated total number of importations and simulated number of countries ever importing mpox clade Ib

Assuming that the risk of importing a clade Ib mpox case from countries with ongoing spread is proportional to the flight travel volume, we simulated the cumulative number of imported cases outside most affected countries before a given country i experiences its first imported case: D_i. That is, (D_i+1)-th imported case outside of countries with an ongoing outbreak would be the first imported case in country i. Similarly, we also simulated the number of countries ever importing mpox clade Ib before the first importation in country i: N_i. Country i would be the (N_i+1)-th country to ever import a case.

Under the assumption that either (i) DRC, (ii) DRC and Burundi and (iii) DRC, Burundi, Uganda, Kenya and Rwanda are the primary source of importation throughout the time course of interest (i.e. we did not consider established local transmission in any other country onward that could contribute to importation elsewhere), we modelled D_i to follow a geometric distribution:

$$D_i \sim \mathrm{Geom}(r_i), \tag{S5}$$

where r_i is the relative flight travel volume among the countries at risk of importation (all countries except the countries which have reported clade Ia or Ib). Given the cumulative number of imported cases globally before country i observed an importation (D_i), the number of countries with nonzero importations before country i, N_i , can be sampled from

$$N_i = \sum_{j \neq i} \left[1 - \delta\left(d_j\right)\right], \ \mathbf{d}_{-i} \sim \mathrm{Multinom}(D_i, \mathbf{r}_{-i}), \tag{S6}$$

where d_j is the cumulative number of imported cases in country j (conditioned on D_i), δ is the Kronecker delta, $\mathbf{d}_{-i}$ is a vector of d_j for all countries except country i, and $\mathbf{r}_{-i}$ is a vector of relative flight travel volume excluding country i. We simulated the model 10,000 times for each assumption and calculated the median, 2.5h and 97.5th percentiles.

The analysis for back-projection was performed in Julia v1.7.3 and the simulation analysis was performed in R v4.1.3. The code used in the present study was deposited at https://github.com/toshiakiasakura/mpox1b_importation_risk.

Supplementary results

Back-projected cumulative incidence in countries with ongoing outbreaks

Based on the WHO situation report as of 29 August 2024,[4] the reported numbers of confirmed and suspected mpox cases in the North and South Kivu provinces—where clade Ib is known to circulate in the DRC—are 1443 and 5007, respectively.

Table S1. Estimated cumulative incidence of mpox clade Ib for each assumption of the primary source of importation.

Date	Cumulative reported cases	T_d (days)	Estimated cumulative incidence (95% confidence interval) for the primary source of importation		
			DRC	DRC, Burundi	DRC, Burundi, Rwanda, Uganda, Kenya
2024-08-15	Sweden (1)	7	11,591 (634-51,624)	11,639 (637-51,839)	3,614 (198-16,095)
		10	8114 (444-36,137)	8148 (446-36,287)	2,530 (138-11,266)
2024-08-22	Sweden (1), Thailand (1)	7	23,183 (3,765-72,255)	23,279 (3,781-72,556)	7,227 (1,174-22,527)
		10	16,228 (2,636-50,579)	16,295 (2,647-50,789)	5,059 (822-15,769)

Sensitivity analysis for the simulated number of importations and number of countries ever importing given one observation in a specific country

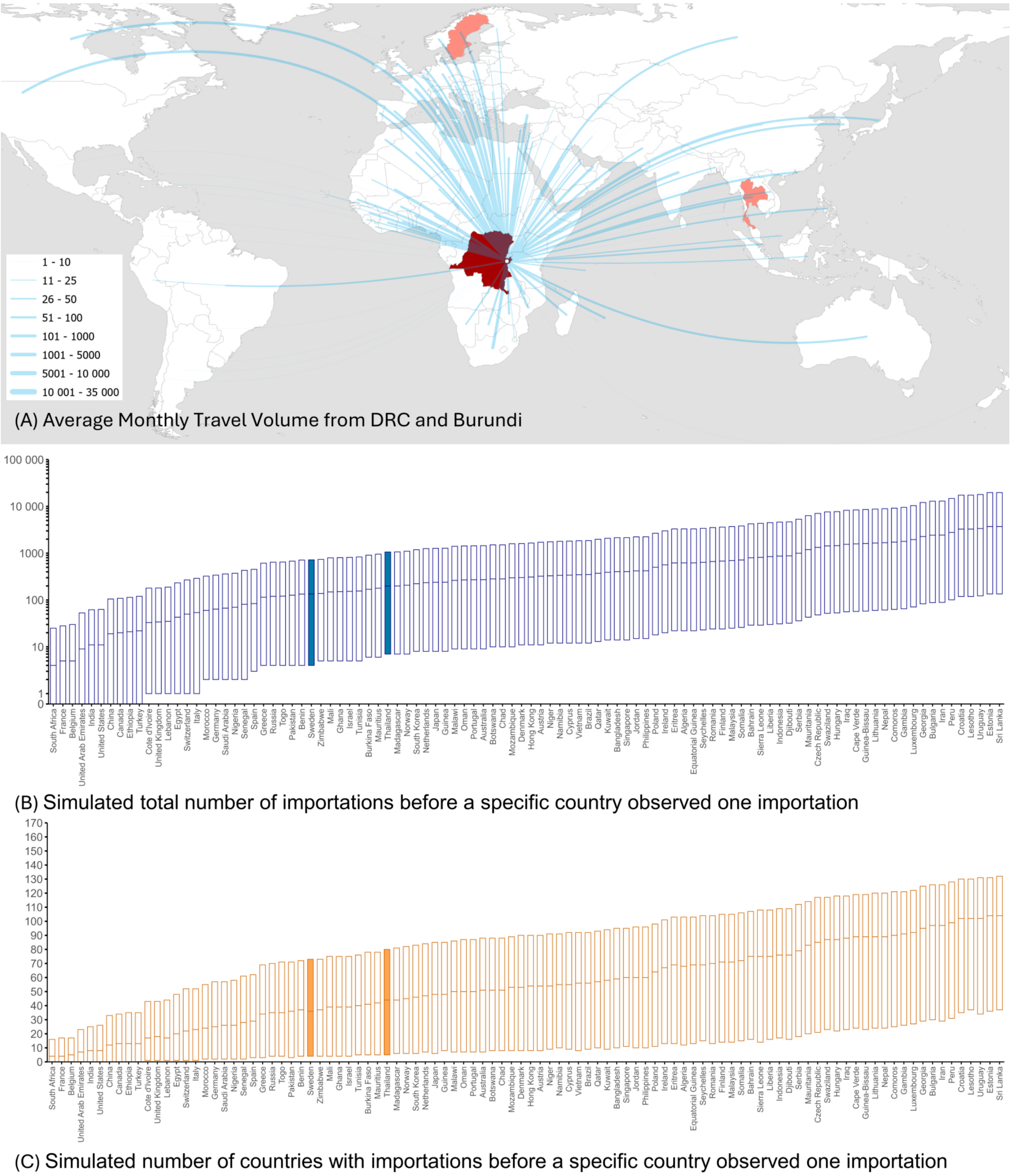

Figure S1. Travel volumes, and simulated number of importations and countries before the first importation in a specific country. (A) Travel volume from the DRC and Burundi combined to international countries. **(B)** Simulated total number of importations before a specific country observed one importation and **(C)** Simulated number of countries with importations

before a specific country observed one importation. Only the top 100 countries with the highest travel volume are included in the figure. Flight volumes for the above countries to Angola, Burundi, Cameroon, DRC, Central African Republic, Gabon, Kenya, Rwanda, Republic of the Congo, South Sudan, Tanzania, Uganda and Zambia were excluded as they have reported historic or current clade Ia or Ib mpox cases.

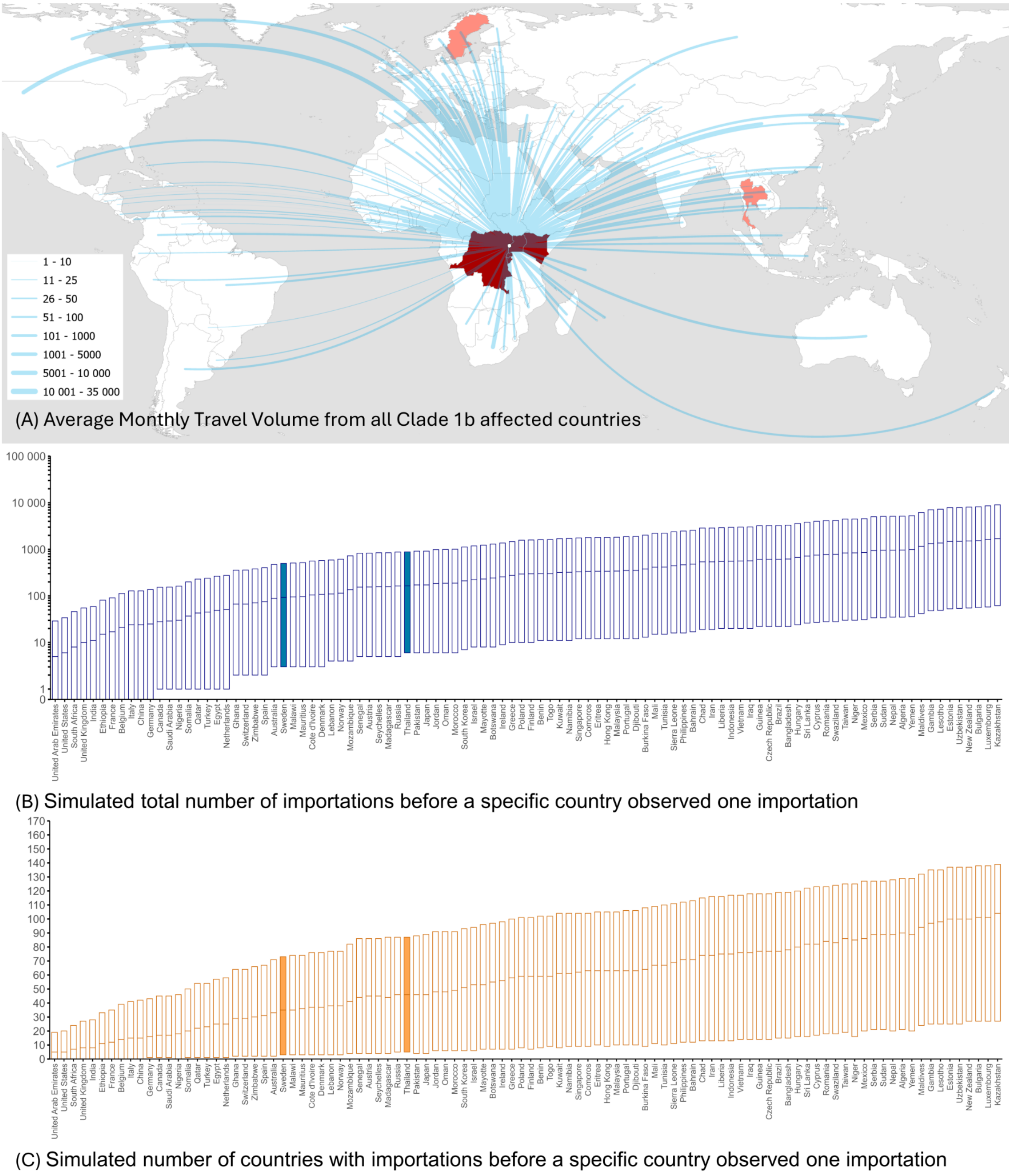

Figure S2. Travel volumes, and simulated number of importations and countries before the first importation in a specific country. (A) Travel volume from the DRC, Burundi, Uganda, Kenya and Rwanda combined to international countries. **(B)** Simulated total number of importations before a specific country observed one importation and **(C)** Simulated number of countries with importations before a specific country observed one importation. Only the top 100 countries with the highest travel volume are included in the figure. Flight volumes for the above countries to Angola, Burundi, Cameroon, DRC, Central African Republic, Gabon, Kenya,

Rwanda, Republic of the Congo, South Sudan, Tanzania, Uganda and Zambia were excluded as they have reported historic or current clade Ia or Ib mpox cases.

References

1 OAG. Flight database & statistics | Aviation analytics | OAG. https://www.oag.com (accessed 6 Sep 2024).

2 United Nations. United Nations | Data Portal Population Division. https://population.un.org/dataportal/home (accessed 6 Sep 2024).

3 GOV.UK. Operational mpox HCID (Clade I) case definition. GOV.UK. https://www.gov.uk/guidance/operational-mpox-monkeypox-hcid-case-definition (accessed 6 Sep 2024).

4 WHO. Rapport de la situation épidémiologique de la variole simienne (Mpox) en RDC sitrep No 025 (29 août 2024) - Democratic Republic of the Congo | ReliefWeb. 2024. https://reliefweb.int/report/democratic-republic-congo/rapport-de-la-situation-epidemiologique-de-la-variole-simienne-mpox-en-rdc-sitrep-no-025-29-aout-2024 (accessed 6 Sep 2024).
